# Dimension reduction with gene expression data using targeted variable importance measurement

**DOI:** 10.1186/1471-2105-12-312

**Published:** 2011-07-29

**Authors:** Hui Wang, Mark J van der Laan

**Affiliations:** 1 Department of Pediatrics, Stanford University, MSOB X111, Stanford, CA 94305, USA; 2 Division of Biostatistics, University of California Berkeley, 101 Haviland Hall, Berkeley, CA 94720, USA

## Abstract

**Background:**

When a large number of candidate variables are present, a dimension reduction procedure is usually conducted to reduce the variable space before the subsequent analysis is carried out. The goal of dimension reduction is to find a list of candidate genes with a more operable length ideally including all the relevant genes. Leaving many uninformative genes in the analysis can lead to biased estimates and reduced power. Therefore, dimension reduction is often considered a necessary predecessor of the analysis because it can not only reduce the cost of handling numerous variables, but also has the potential to improve the performance of the downstream analysis algorithms.

**Results:**

We propose a TMLE-VIM dimension reduction procedure based on the variable importance measurement (VIM) in the frame work of targeted maximum likelihood estimation (TMLE). TMLE is an extension of maximum likelihood estimation targeting the parameter of interest. TMLE-VIM is a two-stage procedure. The first stage resorts to a machine learning algorithm, and the second step improves the first stage estimation with respect to the parameter of interest.

**Conclusions:**

We demonstrate with simulations and data analyses that our approach not only enjoys the prediction power of machine learning algorithms, but also accounts for the correlation structures among variables and therefore produces better variable rankings. When utilized in dimension reduction, TMLE-VIM can help to obtain the shortest possible list with the most truly associated variables.

## Background

Gene expression microarray data are typically characterized by large quantities of variables with unknown correlation structures [1,2]. This high dimensionality has presented us challenges in analyzing the data, especially when correlations among variables are complex. Including many variables in standard statistical analyses can easily cause problems such as singularity and overfitting, and sometimes is not even doable. To manage this problem, the dimensionality of the data will often be reduced in the first step. There are multiple ways to achieve this goal. One is to select a subset of genes based on certain criteria such that this subset of genes is believed to best predict the outcome. This gene selection strategy is typically based on some univariate measurement related to the outcome, such as t-test and rank test [3,4]. Another strategy is to use a weighted combination of genes of lower dimension to represent the total variation of the data. Representative approaches are principle component analysis (PCA) [5] and partial least squares (SLR) [6-9]. Machine learning algorithms such as LASSO [10,11] and Random Forest [12] have embedded capacity to select variables while simultaneously making predictions, and can be used to accommodate high dimensional microarray data.

As always, there is no one-size-fits-all solution to this problem, and one often needs to resort to a mix-and-match strategy. The univariate-measurement based gene selection is a very popular approach in the field. It is fast and scales easily to the dimension of the data. The output is usually stable and easy to understand, and fulfills the objectives of the biologists to directly pursue interesting findings. However, it often relies on over-simplified models. For instance, the univariate analysis evaluates every gene in isolation of others, with the unrealistic assumption of independence among genes. As a result, it carries a lot of noise and the selected genes are often highly correlated, which themselves create problems in subsequent analysis. Also, due to the practical limit of the size of the gene subset, real informative genes with weaker signals will be left out. In contrast, PCA/PLS constructs a few gene components as linear combinations of all genes in a dataset. This "Super Gene" approach assumes that the majority of the variation in the dataset can be explained by a small number of underlying variables. One then uses these gene components to predict the outcome. These approaches can better handle the dependent structure of genes and their performances are quite acceptable [13]. But it is harder to interpret gene components biologically, and to assess the effect of individual genes one needs to look at the weight coefficients of the linear combination. Machine learning algorithms are very attractive variable selection tools to deal with large quantities of genes. They are prediction algorithms with embedded abilities to select gene subsets. However, whether or not a gene is chosen by a learning algorithm may not be the best measurement of its importance. Machine learning algorithms are constructed to achieve an optimal prediction accuracy, which often overlooks the importance of each variable. Consequently, small changes in data or tuning parameters may result in big changes in variable rankings and the the selected gene subsets are instable. For example, Random Forest, a tree-based non-parametric method, has a variable importance measurement that greatly contributes to its popularity. This measurement is sensitive to the parameter choices of trees in the presence of high correlations among variables, because different sets of variables can produce nearly unchanged prediction accuracy [14,15]. Another example is LASSO -- one of the most popular regularization algorithms. Assuming a sparse signal, LASSO handles the high dimensionality problem by shrinking the coefficients of most variables towards zero [16]. A recent implementation of LASSO is in the GLMNET R package [17]. The package uses a coordinate descent algorithm and can finish an analysis of 20,000 variables within a few seconds. To us, its result is somewhat sensitive to the choice of the penalizing parameter *λ*. Different *λ*s may result in gene subsets with little overlapping. In the mean time, variable importance measurements are not readily available in LASSO. One can simply rank genes by their coefficients, but this can be quite subtle. Although permutation tests may be used to derive p-values, how to perform the permutation is a tricky matter due to selection of tuning parameters. For small p-values, it is still computationally infeasible. In this paper, inspired by concepts of counterfactual effects from the causal inference literature, we propose a targeted variable importance measurement [18,19] to rank genes and reduce the dimensionality of the dataset. Counterfactuals are usually defined in the context of treatment to disease. It is the outcome a patient would have had a treatment been assigned differently, with everything else held the same. Hence counterfactuals are "counter"-fact and apparently impossible to be observed. But it can be estimated statistically. Suppose that we have an outcome *Y*, a binary treatment *A*, and the confounding variables *W *of *A*, and we have worked out correctly an estimate  of the conditional expectation of *Y *given *A *and *W*. A common way to estimate the counterfactual effect of *A *is to compute the difference between the  and the  for every observation and then average over all observations, referred to as the G-computation method [20]. Although counterfactuals may not be completely relevant to gene microarray data, thinking about the data in this way is very helpful for us to assess the importance of a gene. Our VIM definition uses the concepts of counterfactuals and the estimation framework is built on the methodology of targeted maximum likelihood estimation (TMLE) [21]. By tailoring this recently developed technique specifically to gene expression data, we hope to introduce to the community an alternative strategy to carry out gene selection in addition to current methods. Our approach takes the advantage of prediction power of learning algorithms while targeting at the individual importance of each variable. Its mathematical property has been studied in [22], and we will focus on its application. In brief, our approach consists of two-stages. In the first stage, we predict the outcome given all genes. In the second stage, we improve the first stage by modeling the mechanism between an individual gene and its confounding variables. Both stages can be very flexible ranging from using univariate analysis to refined learning algorithms. When machine learning algorithms are used, we have the flexibility to determine how to make predictions without restricting ourselves to explicit models and distributions. In the meanwhile, as in the case of the univariate analysis, we return to a simple and well interpretable measure of the importance of each gene. This importance measurement is derived in the presence of the confounding variables of a gene, and hence can help to exploit the redundant information among correlated genes. It is generally also more stable than the variable importance produced by machine learning algorithms. In addition, our approach provides a simple way for statistical inference based on asymptotic theories, and is well suited for the exploratory analysis of microarray data.

## Methods

Suppose the observed data are i.i.d. *O_i _*= (*Y_i_*, *A_i_*, *W_i_*), where *Y *is a continuous outcome, *A *is the gene of interest, *W *is a set of confounding variables of *A*, and *i *= 1, ⋯, *n *indexes the observation. Let Ψ(*a*) represents the variable importance measurement (VIM) of *A*. One can define the VIM of *A *as the marginal effect of *A *on the outcome *Y *at value *A *= *a *relative to *A *= 0 adjusted for *W*, and then averaged over the distribution of *W *[18]:

Consider the semiparametric regression model:

where *f*(*W*) is a function of *W*. With this parameterization, we have Ψ(*a*) = *βa*. We can then view *β *as an index of the VIM of *A*. In the above model, the only assumption we make is the linearity of *A*. The definition of the VIM of *A *is closely related to the definition of the counterfactual effect in causal inference [23]. Although *β *can not be directly interpreted as an causal effect without proper assumptions [19], it serves well as a surrogate of the magnitude of the causal relationship between the outcome and a gene. The motivation of this parameterization is that by selecting more causally related genes, the resulting prediction function will be better generalized to new experiments with the same causal relation between the outcome *Y *and *A*, but a different joint distribution of *W*. If in a next experiment, the technology or the sampling population is somewhat different, but the causal mechanism is still the same, then a prediction function that uses the correlates of the true causal variables will perform poorly while a prediction function using the true causal variables will still perform nicely. This idea will be illustrated in our simulations.

Our goal is to estimate *β*. In [22], this estimation problem was addressed in the framework of targeted maximum likelihood estimation (TMLE). TMLE is an estimating equation and efficient estimation theory based methodology [24], and is particularly useful when it comes to semiparametric models. Estimators from the traditional method such as MLE perform well for parametric models, however, they are generally biased relative to their variances especially when the model space is large. This is because the MLE focuses on doing a good job on the estimation of the whole density rather than on the parameter itself. TMLE is designed to achieve an optimal trade-off between the bias and the variance of the estimator. It uses an MLE framework, but instead of estimating the overall density, TMLE targets on the parameter of interest and produces estimators minimally affected by changes of the nuisance parameters in a model. In Additional File 1 we provide a brief overview of this methodology with a demo simulation example. The formal mathematical formulation of TMLE can be found in the original paper by van der Laan and Rubin [21]. The implementation of TMLE to estimate *β *is fairly simple and consists of two stages. First, we estimate *E*(*Y *|*A*, *W*) without any parametric restriction. We then regress the residual of *Y *and *E*(*Y *|*A *= 0, *W*) onto *βA *to conform with our semiparametric regression model. This will yield an initial estimator of *β *and fitted values of *E*(*Y *|*A*, *W*), denoted by  and the . In the second stage, we update these initial estimates in a direction targeted at *β*. This involves regressing the residuals of *Y *and the fitted  on the clever covariate *A - E*(*A*|*W*). The *E*(*A*|*W*) evaluates the confounding of *A *with *W*, and we name it the "gene confounding mechanism". It needs to be estimated if unknown. Let us denote the coefficient before the clever covariate as *ε*. The updated TMLE estimate of *β *is , where *ε_n _*is the estimated value of *ε*. The variance estimate of *β *can be computed from its efficient influence curve. Below is a step-by-step implementation of our algorithm, and we refer to it as the TMLE-VIM procedure.

1. Obtain the initial estimator  and . Use your favorite algorithm here, for example, linear regression, LASSO, Random Forest, etc.

2. Obtain the *g_n_*(*W*) estimate for the gene confounding mechanism *E*(*A*|*W *). As in the case of *Q_n_*(0), a broad spectrum of algorithms can be used. In this paper, we use LASSO (in the GLMNET R package) for its optimal speed.

3. Compute the "clever covariate":

4. Fit regression .

5. Update the initial estimate  with

and update the initial fitted values  with

6. Compute the variance estimate  for  according to its efficient influence curve:

where *i *indexes the *i*-th observation.

7. Construct the test statistic:

*T*(*A*) follows the standard Gaussian distribution under the null hypothesis *β *= 0 when the sample size *n *goes to infinity.

The TMLE estimator  is a consistent estimator of *β *when either the  or the *g_n_*(*W *) is consistent. When the  is consistent, it is also asymptotically efficient. The derivations of the clever covariate, the efficient influence curve, the TMLE estimate and its mathematical properties can be found in [22] and [18]. Upon the construction of the test statistic, a p-value can be calculated for the adjusted marginal effect of *A *and used as an index of the variable importance.

In the application to dimension reduction, for each variable in the dataset, we compute a TMLE-VIM p-value. We then reduce our variable space based on these p-values. There are two notions. First, in principle, a separate initial estimator  should be fitted for every gene *A *by forcing *A *as a term in the algorithm used. This can become quite time consuming. To solve the problem, instead of estimating *E*(*Y *|*A*, *W*) for each *A*, we obtain a grand estimate *G_n_*(*V*) for *E*(*Y *|*V *). Here *V *represents all variables in the dataset. Then for every *A *in *V*, we carry out the regression *Y *~ *βA *with the offset *G_n_*(*A *= 0) to get *β_n_*(0) and . Second, when obtaining the *g_n_*(*W*), we want to be attentive to how closely *W *is correlated with *A*. The independence between *W *and *A *results in zero adjustment to the initial estimator, while a complete association causes *β *to be unidentifiable. A simple option is to use all variables less than a pre-defined correlation with *A *as *W*. In [22], they authors suggest 0.7 as a conservative threshold. Instead of applying a universal cutoff, we can also set individualized correlation threshold for each *A*. Below we provide a data adaptive procedure to do it. One first defines a sequence of correlation cutoffs *δ*. For each choice of *δ*, one computes the corresponding TMLE p-value for *A*. One then sets a p-value threshold *λ*, and chooses the maximum *δ *that has produced a p-value less than *λ*. The degree of the protection is determined with the value of *λ*. In general, the smaller the *λ *is, the more the protection. The value of *λ *can be either fixed a priori or chosen by cross validation. We refer to it as the TMLE-VIM(*λ*) procedure. It allows us to adjust for the confounding in the dataset adaptively and flexibly, and protect the algorithm against the harm from high correlations among variables. It works best when many variables are closely correlated in a complex way. However, it does require more time to run, especially when *λ *needs to be chosen by cross validation. In many cases, a universal cutoff of 0.7 will work fine. In Additional File 1 we provide the mathematical formulation of the TMLE-VIM(*λ*) procedure. Once we have all the variables ranked by their p-values. the candidate list can be truncated by either applying a p-value threshold or taking the top *k *ranked variables. Both of them are sound practices. In our simulations and data analysis, we usually truncate the list at a p-value threshold 0.05.

## Results and Discussion

### Simulation studies

We performed two sets of simulations. The first set of simulations investigates how TMLE-VIM responds to changes in the number of confounding variables, the correlation level among variables, and the noise levels. The second set studies the TMLE-VIM with more complex correlation structures and model misspecification. The performance of the dimension reduction procedure was primarily evaluated by the achieved prediction accuracy using a prediction algorithm on the reduced sets of variables, illustrated in the following analysis flow:

Two prediction algorithms, LASSO and D/S/A (Deletion/Substitution/Addition) [25], were used. D/S/A searches through the variable space and selects the best subset of covariates by minimizing the cross validated residual sum of squares. In our simulations, LASSO and D/S/A predictions are often similar. We used D/S/A in simulation I as it provides convenience to count what variables are included in the prediction model. LASSO was used in simulation II for its faster speed. We also used multivariate linear regression (MVR) as a comparison to machine learning algorithms when applicable.

#### Part I

In simulation I, we varied the number of non-causal variables (*W*), the correlation coefficient *ρ *among variables, and the noise level  to see how TMLE-VIM responds to them. For each simulated observation *O_i _*= (*Y_i_*, *A_i_*, *W_i_*), where *i *indexes the *i*-th sample, the outcome *Y_i _*was generated from a main effect model of 25 *A*s:

where *j *indexes the *j*-th *A*, and *e_i _*is a normal error with mean 0 and variance . Each *A_j _*was correlated with *m W*s, and hence the total number of *W*s is *m_w _*= 25 *m*. *A_j _*and its associated *W *s were jointly sampled from a multivariate Gaussian distribution with mean 0 and variance-covariance matrix *S*, where *S *is a correlation matrix with an exchangeable correlation coefficient *ρ*. This simulation scheme resulted in 25 independent clusters of covariates. Within each cluster, the covariates are correlated at level *ρ*.

Simulations were run for combinations of:

• *m *= (10, 20) corresponding to *m_w _*= (250, 500);

• *σ_e _*= (1, 5, 10);

• and *ρ *= (0.1, 0.3, 0.5, 0.7, 0.9).

For each combination, we simulated a training set of 500 data points and a testing set of 5000 data points. The training set was used to obtain the prediction model while the testing set was used to calculate the L2 risk. We also calculated a cross-examined L2 risk using a testing set with a *ρ *other than that of the training set. This is to demonstrate that by identifying more causally related variables, TMLE-VIM is robust to the change of the joint distribution among the covariates *A*s and *W*s. In specific, for each prediction model obtained from a training set, we calculated the *L*2 risk on the testing set generated with *ρ *= 0.1 regardless of what *ρ *was used to generate the training set. As a benchmark, we also used univariate regression in parallel with TMLE-VIM to reduce the dimensionality of the dataset, denoted with UR-VIM. Once the variable importance was calculated, we cut short the variable list using a p-value threshold 0.05. Each combination was replicated 10 times and results took the average.

TMLE-VIM used LASSO to obtain both the initial estimator  and the gene confounding mechanism estimator *g_n_*(*W *). In the *g_n_*(*W *), *W *was all the variables excluding *A*. TMLE-VIM has demonstrated a consistent advantage over UR-VIM with respect to the final prediction error over a range of simulation settings. This is particularly the case when the joint distribution of the covariates changes and when predictions were made by MVR that lacks internal capacity of model selection. Smaller *σ_e_*, larger *m_w_*, and larger *ρ *tend to magnify this advantage. Also, TMLE-VIM risks have smaller standard errors than the UR-VIM risks. In Table 1, we present our simulation results for five different *ρ *values and two different *m_w _*values, with  fixed at 5. The following summary quantities are reported:

**Table 1:** The simulation I results

*ρ*		*m_w _*= 250		*m_w _*= 500	
			
		MVR	DSA	MVR	DSA
	*R_r_*	**0.2341 **; *0.2251*	**0.2436 **; *0.2351*	**0.4035 **; *0.3784*	**0.4230 **; *0.3943*
0.1	*R_A_*	1.0870	-	1.2136	-
	*R_W_*	0.6522	-	0.8846	-
	*RR_DSA_*	na	1.0130	na	1.0680
	*R_r_*	**0.2202 **; *0.2297*	**0.2231 **; *0.2247*	**0.2341 **; *0.2307*	**0.2027 **; *0.1975*
0.3	*R_A_*	1.0776	-	1.0684	-
	*R_W_*	0.1528	-	0.0958	-
	*RR_DSA_*	na	1.0345	na	1.0299

	*R_r_*	**0.2425 **; *0.3115*	**0.1169 **; *0.1285*	**0.4883 **; *0.5959*	**0.1268 **; *0.1217*
0.5	*R_A_*	1.0373	-	1.0331	-
	*R_W_*	0.0355	-	0.0149	-
	*RR_DSA_*	na	1.0251	na	1.0335

	*R_r_*	**0.3599 **; *0.5872*	**0.1307 **; *0.2545*	**0.8001 **; *0.9093*	**0.1740 **; *0.2976*
0.7	*R_A_*	1.0081	-	1.0000	-
	*R_W_*	0.0275	-	0.0162	-
	*RR_DSA_*	na	1.0693	na	1.1055

	*R_r_*	**0.2262 **; *0.7248*	**-0.1364 **; *0.2805*	**0.9390 **; *0.9885*	**-0.5498 **; *0.2657*
0.9	*R_A_*	0.8415	-	0.5502	-
	*R_W_*	0.0364	-	0.0204	-
	*RR_DSA_*	na	1.2630	na	1.6103

Bold fonts: testing set (a). Italic fonts: testing set (b).

na: not available. -: the same value as the previous entry.

• *R_r _*= (UR-VIM risk *- *TMLE-VIM risk)*/*UR risk: the proportion of the risk reduction of TMLE-VIM relative to the UR-VIM risk. It measures by how much TMLE-VIM outperforms UR-VIM. The bigger the number, the more the advantage.

•*R_A _*= TMLE-VIM *N_A_/*UR-VIM *N_A_*: the ratio of the number of *A*s (*N_A_*) in the TMLE-VIM list to the number of *A*s in the UR-VIM list.

•*R_W _*= TMLE-VIM *N_W_/*UR-VIM *N_W _*: the ratio of the number of *W*_s _(*N_W_*) in the TMLE-VIM list to the number of *W*s in the UR-VIM list.

•*RR*_DSA _= TMLE-VIM *P_A_/*UR-VIM *P_A_*: the ratio of the proportion of *A*s (*P_A_*) in the final D/S/A prediction model resulted from the TMLE-VIM procedure to that from the UR-VIM. It measures the relative chance of arriving at a truly associated variable in the final model through the path of TMLE-VIM, referenced to the UR-VIM.

The *R_r _*was calculated on two different testing sets. One is the testing set generated with the same *ρ *as the corresponding training set, and we refer it to "testing set (a)"; the other is the testing set generated with *ρ *= 0.1, and we refer it to "testing set (b)". Testing set (a) shares the same correlation structure as the training set, while in testing set (b) all the variables are essentially independent of each other. Testing set (b) is a simple representation of the scenario that when a new experiment is conducted the overall joint distribution of the covariates changes while the causal mechanism remains the same. In Table 1, the bold *R_r _*was calculated on testing set (a), and the Italic *R_r _*was on testing set (b). We make a few points here about Table 1:

• The proportion of the risk reduction (*R_r_*) of the TMLE-VIM relative to the UR-VIM is typically more than 20% for the MVR prediction and 10% for the D/S/A prediction. In some cases, the risk reduction of the MVR can be very significant. For example, when *m_w _*= 500 and *ρ *= 0.7, the TMLE-VIM risk is close to only half of the UR-VIM risk. TMLE-VIM tends to deliver more advantages when *m_w _*= 500 than when *m_w _*= 250. When the correlation coefficient *ρ *increases, the TMLE-VIM performs increasingly better than the UR-VIM for the MVR prediction. For the D/S/A prediction, small or large *ρ*s seem to benefit most from the TMLE-VIM. For intermediate *ρ*, the benefit is still there but reduced. We believe that how much the risk can be reduced by the TMLE-VIM is a combination of factors such as the number of *A*s and *W*s in the reduced candidate list, the correlation structures among covariates and the internal optimization procedures of D/S/A. The advantage of the TMLE-VIM over the UR-VIM does seem to be more significant on the testing set (b) than the testing set (a), in support of our hypothesis that by identifying more causally related variables the TMLE-VIM results generalize better to new experiments.

• Most *R_A _*values are slightly higher than 1 while the *R_W _*values are much smaller. This indicates that on average, in the TMLE-VIM list, the number of correctly identified *A*s is slightly higher than that in the UR-VIM list, while the number of falsely associated *W*_s _is much less. It is especially the case when the correlations are high among variables. The low counts of false positives is a major contributing factor that the prediction made on the TMLE-VIM candidate list is better than that on the UR-VIM.

• As to the number of *A*s that are finally made into the D/S/A prediction model, the TMLE-VIM in most cases displays a slight advantage over the UR-VIM. A closer look reveals that the variables included in the D/S/A model only differs by one or two between the TMLE-VIM and the UR-VIM. But the prediction risk has a measurable difference. This probably implies that every single variable counts in making good predictions in these simulations.

• When *ρ *= 0.9, the situation seems to be losing its track. The TMLE-VIM did worse than the UR-VIM in terms of correctly identified variables as well as the prediction risk of the testing set (a). Considering the high correlations among variables, this could possibly be attributed to the overfitting in the *g_n_*(*W *). Indeed, in [22], the authors showed that TMLE deteriorates when adjusting for variables with correlation coefficients beyond 0.7. However, the *RR_DSA _*indicates that the chance of including a correct variable in the final D/S/A model based on the TMLE-VIM list is higher than that on the UR-VIM. Further looking into the data, we found out that the number of *A*s that made into the D/S/A model from the TMLE-VIM list is actually greater than that from the UR-VIM, while the number of *W*s is much less. Henceforth, the D/S/A model built on the TMLE-VIM list is closer to truth, but somehow its prediction is worse than the model built on the UR-VIM list. This seems to suggest that when provided with the UR-VIM list, the D/S/A has offset its model for the missed *A*s from highly correlated *W*s, while for the TMLE-VIM, this can not be done since there are not many *W*s in the list. It is the same reason that the UR-VIM underperforms the TMLE-VIM on the testing set (b) when those surrogates of *A*s were lost. For the MVR, although the TMLE-VIM shows a dominant advantage over the UR-VIM with respect to the prediction accuracy, the TMLE-VIM only identified 77% (when *m_w _*= 250) and 57% (when *m_w _*= 500) of the *A*s identified by the UR-VIM. The better prediction is merely due to the fact that the MVR breaks down when too many variables entered the model. This is particularly the case when *m_w _*= 500.

**Figure 1:**
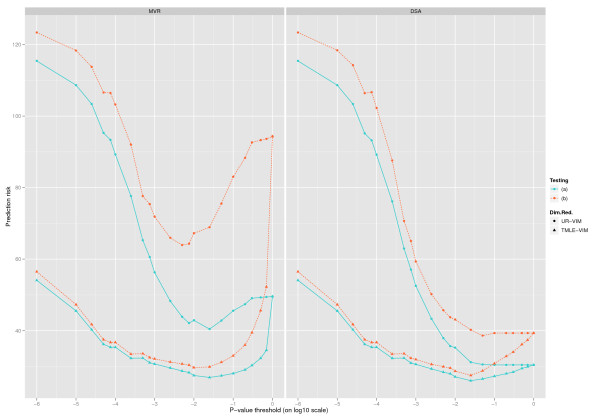
**A typical example in simulation I**. This graph presents the average L2 risk of the final prediction model on the candidate list from the UR-VIM and the TMLE-VIM, for simulation I data with setup (*σ_e _*= 5, *m_w _*= 10, *ρ *= 0.7). In the left panel, the MVR risk is plotted against a series of p-value thresholds used to truncate the candidate list; the right panel plots the D/S/A risk. The testing set (a) predictions are grouped in solid blue lines, and the testing set (b) predictions are grouped in broken orange lines. Dots represent the UR-VIM, and triangles represent the TMLE-VIM.

Figure 1 presents a graphical representation of a typical example in simulation I with (*σ_e _*= 5, *m_w _*= 250, *ρ *= 0.7). Besides the advantage displayed by the TMLE-VIM relative to the UR-VIM, we also see much smaller differences between the TMLE-VIM risks of the testing set (a) and (b) compared to the UR-VIM because TMLE-VIM was able to detect more *A*s. In summary, when confounder are properly adjusted in *g_n_*(*W *), TMLE-VIM improves not only the performance of relatively simple algorithms such as the MVR, but also the more complex learning algorithms with built-in capacities of variable selection. Interested readers can find all the original prediction risks and counts of *A*s and *W*s and their standard errors in Additional File 2 for this simulation.

#### Part II

Simulation II examines the TMLE-VIM on larger-scale datasets with much more complex correlation structures. The simulation consists of 500 samples and 1000 variables. We used a correlation matrix derived from the top 800 genes in a real dataset published in [26]. For these genes, the median absolute correlation coefficient was centered at 0.26, the 1st/3rd quartile being 0.16/0.37, and the maximum as high as 0.9977. Hence, simulation II tried to mimic the correlation structure in this real data set. The outcome *Y *was generated from two different models using 20 *A*s. One is a linear model, and the other is polynomial.

Details of this simulation is provided in the Additional File 1. A test dataset of 5000 points were simulated to assess the L2 prediction risk. We repeated the simulation for 10 times and results took the average. In TMLE-VIM, we tried two different initial estimators. One is the univariate regression as simple as *Y ~ A*, and the other is the LASSO estimator. LASSO was also used to get the *g_n_*(*W *) and to make the final predictions. we adjusted universally in the *g_n_*(*W *) for the variables that are correlated with *A *with an arbitrary correlation coefficient less than 0.7. All the  and *g_n_*(*W *) models were main-term linear. Hence, with the polynomial outcome, we could examine how TMLE-VIM performs when mis-specified models were provided. To summarize the result, we computed a *R*^2 ^quantity, representing the proportion of explained variance relative to an intercept model. It is defined as 1 - mean risk/MST, where  and  is the mean of *Y*. Table 2 lists the *R*^2 ^and the number of true positives (T.P.) and false positives (F.P.) in the reduced list of candidate variables, for the UR-VIM, the , and the . Compared to UR-VIM, TMLE-VIM improved the prediction risk by providing LASSO a candidate list with more truly and less falsely associated variables for both the linear and polynomial simulations. It is worth noting that even with an initial estimator as simple as the univariate regression , TMLE-VIM still achieves a significant increase in *R*^2 ^by modeling the *g_n_*(*W *).

**Table 2:** The simulation II results (p-value)

	**Simulation**					
	
	**Linear**			**Polynomial**		
		
	***R*^2^**	**T.P**	**F.P**.	***R*^2^**	**T.P**.	**F.P**.
UR-VIM	0.2887	13.8	605.3	0.1851	13.4	555.9
	0.4849	16.6	280.5	0.3245	14.7	255.5
	0.6289	19.7	29.1	0.4203	17.9	24
TMLE-VIM(*λ*)	0.6479	20	41.6	0.4498	19.2	105.9

The candidate variable list contains all variables with p-values less than 0.05.

The numbers in Table 2 were based on candidate lists that were cut short with a p-value threshold of 0.05. In Table 3, we provide the results based on the top 100 ranked genes. The numbers of UR-VIM and the  are less satisfying than those in Table 2, while the  achieved comparable results. This suggests that the  p-values of *A*s are among the smallest ones, and shortening the length of the list does not affect the final result. Regardless of the weakened results, The  still displays a non-ignorable advantage over the UR-VIM with respect to the prediction accuracy, while the number of correctly identified *A*s is slightly smaller than that of the UR-VIM. We then looked at the correlation matrix among the top 100 selected genes, and it occurs that the correlation among them is the least for the , the most for the UR-VIM, and the  lies in between. This could explain why the  does a better job in prediction regardless of less *A*s.

**Table 3:** The simulation II results (top 100)

	**Simulation**					
	
	**Linear**			**Polynomial**		
		
	***R*^2^**	**T.P**	**cor**.	***R*^2^**	**T.P**.	**cor**.
UR-VIM	0.1444	9.0	0.2956	0.0862	8.2	0.3642
	0.1907	8.8	0.2534	0.1605	7.2	0.2590
	0.6059	19.9	0.2289	0.4132	19.2	0.2234
TMLE-VIM(*λ*)	0.5916	20	0.1242	0.3859	17.7	0.0867

The candidate variable list contains the top 100 variables ranked by their p-values.

We also carried out the TMLE-VIM(*λ*) procedure with LASSO as the initial estimator, allowing the data select the correlation cutoff for variables to be adjusted in the *g_n_*(*W *). Results are also reported in Table 2 and Table 3. TMLE-VIM(*λ*) identified more *A*s but also more *W*s, and the prediction accuracy is only slightly improved. On the other hand, the correlations among the selected top 100 variables are quite small. It seems by data adaptively adjusting for the correlation levels in the *g_n_*(*W*), TMLE-VIM(*λ*) returns a more independent set of genes. The actual risks and standard errors are contained in Additional File 2.

### Data Analysis

Breast cancer patients are often put on chemotherapy after the surgical removal of the tumor. However not all patients will respond to chemotherapy, and proper guidance for selecting the optimal regimen is needed. Gene expression data have the potential for such predictions, as studied in [26]. The dataset from [26] contains the gene expression profiling on 22283 genes for 133 breast cancer patients. The outcome is the pathological complete response (pCR). This is a binary response associated with long-term cancer free survival. There are also 13 clinical variables collected in the dataset including the ER (estrogen receptor) status, which is a very significant clinical indicator for chemotherapy response.

The goal of the study is to select a set of genes that best predict the clinical response pCR. The first step is to reduce the number of genes worth of consideration, and we applied both UR-VIM and TMLE-VIM (with *Q*^(0) ^= *UR *and *Q*^(0) ^= LASSO) for this purpose. For the TMLE-VIM(*Q*^(0) ^= LASSO), the  was estimated by LASSO using the top 5000 ranked genes. We then took all the genes with the FDR-adjusted p-values less than 0.005 [27], as suggested in the original paper, and upon them we built a predictor using the Random Forest (tuning parameters mtry = number of variables/3, ntree = 3000 and nodesize = 1). The clinical covariates were treated in the same way as genes. To prevent the algorithm from breaking down, we only adjusted for the confounder with correlation coefficients less than 0.7 with *A *in the *g_n_*(*W *). We carried out a 10-fold honest cross validation. We divided the dataset into 10 subsets. Each subset was regarded as a validation set and the rest as the training set. We reperformed the entire analysis, i.e. VIM calculation → dimension reduction → Random Forest classifier, on all 10 training sets and predicted the outcome of the validation set using the classifier built on the training samples. We can then use these cross validated predictions to assess the true classification accuracy of our algorithm.

**Figure 2:**
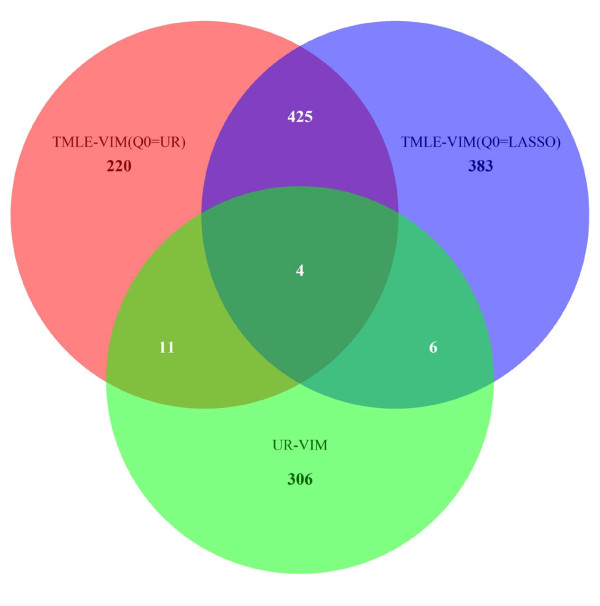
**The venn diagram of the breast cancer data**. This venn diagram shows the overlaps of identified candidate genes from the breast cancer dataset using the UR-VIM, the , and the .

Analysis results are tabulated in Table 4. The UR-VIM produced a candidate list of 326 genes and one clinical variable the "ER status", while the list of the TMLE-VIM(*Q*^(0) ^= UR) consists of 660 genes and TMLE-VIM(*Q*^(0) ^= LASSO) 818 genes. The TMLE-VIM identified many more genes than the UR-VIM. Among all the identified genes, 429 overlap between the  and , 15 overlap between the UR-VIM and TMLE-VIM(*Q*^(0) ^= UR), 10 overlap between the UR-VIM and TMLE-VIM(*Q*^(0) ^= LASSO), and only 4 genes are shared among all three (please see Figure 2). The TMLE-VIM appeared to have selected almost a different set of genes than the UR-VIM.

**Table 4:** The analysis result of the breast cancer dataset

	Num. of genes in the candidate list	C.V. classification accuracy	Corr. level among the top 100 genes
UR-VIM	327	0.7669	0.43
	660	0.7744	0.18
	818	0.7744	0.21

The TMLE-VIM(*Q*^(0) ^= UR) and the TMLE-VIM(*Q*^(0) ^= LASSO) results are quite similar to each other regardless of the adequate difference between the initial estimators. It seems the modeling of the *g_n_*(*W *) had played a significant role and steered away the initial univariate estimates. Further investigation found out that genes in the UR-VIM list are highly correlated with the clinical indicator ER status, while the TMLE-VIM genes are not. Consequently, the TMLE-VIM genes are less correlated to each other than the UR-VIM genes. Looking at the first 100 ranked genes, the absolute median of the correlation coefficients for the UR-VIM is 0.43, while for the TMLE-VIM, it is about half of that number. Although the input variables to the Random Forest are different, The cross validated (CV) classification accuracy are quite similar among these three methods. We also passed all 22,000 genes to Random Forest and looked at its variable importance measurement. The Random Forest VIM (RF-VIM) is more similar to the UR-VIM: about 50% of them overlap but only a few overlap with the TMLE-VIM. The RF-VIM genes are also highly correlated with the ER status, albeit the less severity than the UR-VIM. Its OOB classification accuracy (0.7669) is comparable with all three other methods.

In summary, the UR-VIM and RF-VIM seemed to have identified genes that are strong predictors of the clinical variable ER status. The ER status is a strong indicator of the outcome pCR. Hence, the final prediction accuracy still seems quite good. The TMLE-VIM has identified a list of genes of which a small proportion is strong predictors of ER status and others are not associated with the ER status. Its prediction accuracy is slightly better than that of the UR-VIM and RF-VIM.

## Conclusions

We have shown in this paper with extensive simulations that the TMLE based variable importance measurement can be incorporated into a dimension reduction procedure to improve the quality of the list of the candidate variables. It requires an initial estimator  and a gene confounding mechanism estimate *g_n_*(*W*). A consistent  ensures the consistency and the efficiency of the TMLE estimate. When  is not consistent, a correct specification of *g_n_*(*W*) can still produce consistent estimates while that estimate will not be efficient any more. We generally recommend to do as good a job as we can on obtaining the , as a better  means both a smaller bias and a smaller variance. Nevertheless, algorithms as simple as univariate regression are also valid choices, and in this case, we will rely solely on the goodness of *g_n_*(*W *). The computation of *g_n_*(*W *) directly affects the speed of the TMLE-VIM, as it has to be redone for every variable. Hence, one may want to choose an approach that is reasonably fast. In our study, we chose the GLMNET R Package as our primary tool to get *g_n_*(*W *), and it worked very well. In practice, one needs to balance the resources used for the initial estimator and the gene confounding mechanism. With a proper design of the two estimating stages, TMLE-VIM is a fairly fast procedure. It is also worth mentioning that the TMLE-VIM can sometimes be sensitive to the overfitting in the , and hence, caution needs to be exercised when choosing an aggressive algorithm.

A popular dimension reduction approach is the principle component analysis (PCA). The PCA computation does not involve the outcome, and so it could be less powerful when prediction is the primary goal. Its output is a linear combination of all the genes. Though not a gene selection approach, we still carried it out on our simulation I data as an interesting comparison to our approach. PCA demonstrates an intermediate performance with respect to the UR-VIM and the TMLE-VIM on small p-value cutoffs. This means a few top components carry all the prediction power. When the p-value cutoff is increased, and more components enter the candidate list, its results became quite unsatisfying. When the correlation structure changes among the genes, PCA has done a poor predicting job. The PCA results are contained in Additional File 3.

Usually, the reduced set of variables will serve as the input of a prediction algorithm to build a model. Such algorithms used in this article include MVR, LASSO, and D/S/A. We have noticed that in most of our simulations, the MVR prediction often achieves a similar risk as LASSO and D/S/A on the TMLE-VIM reduced set of variables. It suggests that further variable selection may not be necessary for the TMLE-VIM candidate list, and we can use simpler algorithms to get a good prediction. In fact, the TMLE-VIM can go beyond the scope of dimension reduction. It can be iteratively applied to the data until it converges to a list of several variables that are most likely to be causal to the outcome. In this case, one may want to use the Super Learner [28] as the prediction algorithm, which works more effectively with the TMLE-VIM. The Super Learner is an ensemble learner that combines predictions from multiple candidate learners with optimal weights. It has been shown in [29] that the Super Learner performs asymptotically equal to or better than any of its candidate learners. The Super Learner allows the data to objectively blend results from different algorithms rather than relying on a single algorithm chosen subjectively by an analyst. Hence it enjoys a greater flexibility to explore the model space and usually produces reasonable predictions consistently across a wide variety of datasets, and serves as a very good prediction algorithm for the TMLE-VIM. On the other hand, it is also more computationally demanding.

TMLE-VIM is a quite general approach. Besides gene expression data, TMLE-VIM can also be applied to genetic mapping problems. The genome-wide association studies (GWAS) can involve more than a million of genetic markers. In this case, only the univariate analysis seems to be feasible of ranking every marker. With the TMLE-VIM procedure, we can run more complex algorithms on a subset of top ranked markers, taking it as the initial estimator, and then evaluate every single marker. The variable importance of each marker is thus obtained through a multi-marker approach and being adjusted for its confounder. However, the GWAS in human beings is usually case-control data, and the current TMLE-VIM needs to be extended to accommodate such outcomes.

## Competing interests

The authors declare that they have no competing interests.

## Authors' contributions

MvdL conceived the project and designed the algorithm. HW implemented the algorithm, designed the simulation studies, and collected and analyzed the data. All authors participated in drafting the manuscript.

## Supplementary Material

Additional file 1 **More detailed descriptions of the TMLE methodology and the conducted simulations**. Click here for file

Additional file 2 **The additional materials of the conducted simulations**. Click here for file

Additional file 3 **The PCA results**. Click here for file
