# Supplementary material for: Dimension reduction with gene expression data using targeted variable importance measurement
Source: BMC Bioinformatics. 2011 Jul 29;12:312. doi: 10.1186/1471-2105-12-312 (PMC3166941; doi:10.1186/1471-2105-12-312)
Supplement: Additional file 1 — More detailed descriptions of the TMLE methodology and the conducted simulations. [file 1471-2105-12-312-S1.PDF]

A Brief Overview of TMLE

Suppose $\mathscr{M}$ is a model, p_0 is a density function in $\mathscr{M}$, and $\Psi(p_0)$ is a function that maps p_0 into a vector valued parameter. For a pathwise differentiable parameter $\varphi_0 = \Psi(p_0)$, its pathwise derivative at p_0 can be written as the covariance of the efficient influence curve D^* and the score of the path S:

$$\frac{d}{d\varepsilon}\Psi(p_0(\varepsilon))_{|\varepsilon=0} = E[D^*S].$$

Suppose there exists an initial density estimator $p_n^{(0)}$ based on the empirical probability distribution P_n with the efficient IC $D^*(p_n^{(0)})$. We can find a parametric fluctuation $p_n^{(0)}(\varepsilon)$ indexed by ε through the initial estimator $p_n^{(0)}$, whose score at $\varepsilon = 0$ is equal to the efficient IC, mathematically expressed as:

$$(1) \qquad p_n^{(0)}(\varepsilon = 0) = p_n^{(0)} \quad \text{and} \quad \frac{d\log(p_n^{(0)}(\varepsilon))}{d\varepsilon}_{|\varepsilon=0} = D^*(p_n^{(0)}).$$

This fluctuation function plays the role of stretching the initial density estimator $p_n^{(0)}$ in a direction that targets the parameter of interest. A "good" fluctuation can be found by maximizing the log-likelihood of $p_n^{(0)}(\varepsilon)$ with respect to ε:

$$(2) \qquad \varepsilon_m^{(1)} = argmax_{\varepsilon}\sum_i^n \log(p_n^{(0)}(\varepsilon))(O_i).$$

The 1st step TMLE density estimator $p_n^{(1)}$ for p_0 can then be defined as:

$$p_n^{(1)} = p_n^{(0)}(\varepsilon_m^{(1)}),$$

and the 1st step TMLE estimator $\widehat{\varphi_n}^{(1)}$ for φ_0 is:

$$\varphi_n^{(1)} = \Psi(p_n^{(1)}).$$

This process may need to be iterated to reach convergence.
The observed data is $O = (Y,A,W)$, and the proposed semiparametric model is $E[Y|A,M^-] = \beta A + f(W)$. Let $p(Y|A,W)$ and $p(A|W)$ denote the conditional densities and $p(W)$ the marginal density. The observed data can be factorized into three orthogonal parts:

$$p(Y,A,W) = p(Y|A,W)p(A|W)p(W).$$

The parameter β is only a function of $p(Y|A,W)$. Therefore the efficient IC of β represents only a score for $p(Y|A,W)$, and the targeted fluctuation function can be

selected to only fluctuate the conditional density $p(Y|A,W)$. The efficient IC of β is given below:

$$(3) \quad D^*(p) = \frac{1}{E[A(A-E[A|M^-])]}(A-E[A|M^-])(Y-\beta A - E[Y|A=0,M^-]).$$

Consider the following parametric fluctuation:

$$p(\varepsilon) \propto d_0(Y - \beta_\varepsilon A - \theta_\varepsilon),$$

where d_0 is the standard normal density, $\beta_\varepsilon = \beta + \varepsilon$, and $\theta_\varepsilon = E[Y|A=0,W] - \varepsilon E[A|W]$. It follows that, as required, the score of this fluctuation at $\varepsilon = 0$ spans the efficient IC. The maximum likelihood estimator of ε for this fluctuation function can be obtained through a simple linear regression, in which the residuals from $E[Y|A,W]$ is regressed on the residuals from $E(A|W)$. With this parameterization, TMLE convergence is achieved in one step. When n goes to infinity, we have the asymptotic result:

$$\sqrt{n}(\beta_n - \beta_0) \sim N(0, \sigma_n^2),$$

where $\sigma_n^2 = \frac{1}{n}\sum_{i=1}^n \hat{\text{IC}}(O_i)^2$ is the estimate of the asymptotic variance of β_n. The $\hat{\text{IC}}(O_i)$ is the estimate of the efficient IC and can be obtained by plugging estimates of $E[Y|A,W]$ and $E[A|W]$ in formula 3. One can then carry out a statistical test on β.

A Demo Simulation of TMLE

We simulated 1000 outcomes Y from the following model:

$$Y = 0.5A + 2W_1W_2 + 3W_2W_3^2 - W_1W_3 + e,$$

where $e \sim N(0,10)$. The W_1, W_2, and W_3 were sampled jointly from a multivariate normal distribution. Each W is centered at 0.5 with variance 1. The correlation coefficients are 0.76 between W_1 and W_2, 0.66 between W_2 and W_3, and 0.69 between W_3 and W_1. The variable A was the product of three Ws:

$$A = W_1W_2W_3 + e_A,$$

where e_A is the standard normal error. We are interested in the effect of A on Y, i.e. the β in the semiparametric model $Y = \beta A + f(W)$. To carry out the TMLE, we used a linear regression of Y on A and all three Ws as the initial estimator $Q_n^{(0)}$, i.e.

$$Y \sim A + W_1 + W_2 + W_3.$$

Table 1: the mean estimate of β and its standard error.

	Mean	Standard error
$Q_n^{(0)}$	3.2041	0.2236
TMLE($g_n(W)$=POLYMARS)	0.7233	0.3889
TMLE($g_n(W)$=correct model)	0.5187	0.3573

We used the POLYMARS (the multivariate adaptive polynomial spline regression) in R to model the $g_n(W)$. We also used the correct model for the $g_n(W)$, i.e. $A \sim I(W_1W_2W_3)$. This simulation was repeated for 100 times. The mean estimate of β and its standard error were reported in Table 1. The true value of β is 0.5, and we can see the initial estimate of β from a mis-specified $Q_n^{(0)}$ is highly biased. TMLE was able to correct the bias by modeling $g_n(W)$ at a price of a small increase in the standard error of the estimator. How much the TMLE can correct depends on how good the $g_n(W)$ is, and the correctly modeled $g_n(W)$ has achieved a full bias reduction.

The TMLE-VIM(λ) Procedure

Suppose we have a sequence of correlation cutoffs δ_k, $k = 1, \cdots, K$. For a variable A, one computes its correlation coefficients with all the remaining variables W. Applying δ_k on these correlations identifies a sequence of adjustment sets W_{δ_k}, whose variables are correlated with A at a level less than δ_k. These adjustment sets correspond to a sequence of gene confounding mechanisms $g_n(W_{\delta_k})$, which when applied in TMLE-VIM produces a sequences of TMLE-VIM p-values $p(W_{\delta_k})$. Let λ be a pre-fixed number, the TMLE-VIM(λ) chooses the W_{δ_k} associated with the largest TMLE-VIM p-value $p(W_{\delta_k})$ that are less than λ among all W_{δ_k} as the adjustment set in its gene confounding mechanism. Let $p(\lambda)$ denote the TMLE-VIM(λ) p-value, mathematically, it can be written as:

$$p(\lambda) = p(\delta_h), \text{ where } \delta_h = \text{argmax}_{\delta_k}(p(W_{\delta_k}) < \lambda), \; k = 1, \cdots, K.$$

Below we illustrate the TMLE-VIM(λ) with a simple example. Suppose that we have three variables V_1, V_2, and V_3, with a correlation matrix:

	$V1$	$V2$	$V3$
$V1$	1.0	0.3	0.5
$V2$	0.3	1.0	0.7
$V3$	0.5	0.7	1.0

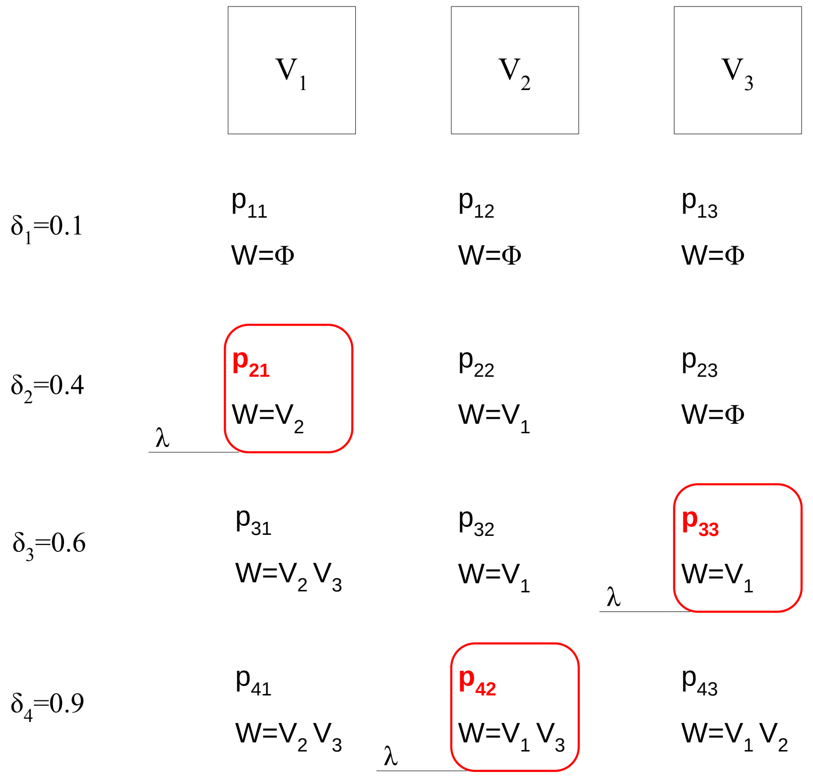

Figure 1: Figure AF1 - The graphical illustration of TMLE-VIM(λ) for the three-variables

For each of these three variables, we compute the TMLE-VIM p-values at four correlation coefficient cutoffs $\delta = (0.1, 0.4, 0.6, 0.9)$. We denote these TMLE p-values as p_{kj}, where k indexes δ_k and j indexes V_j. For variable V_1, suppose both p_{11} and p_{21} are less than λ, and among them, the maximum δ value is $\delta_2 = 0.4$, and therefore $p_1(\lambda) = p_1(\delta_2 = 0.4) = p_{21}$ with adjustment set $W = (V_2)$. Similarly, for V_2, $p_2(\lambda) = p_2(\delta_4 = 0.9) = p_{42}$ with adjustment set $W = (V_1, V_3)$; for V_3, $p_3(\lambda) = p_j(\delta_4 = 0.6) = p_{33}$ with adjustment set $W = (V_1, V_2)$. Figure AF1 shows how the adjustment set is defined for each variable at every δ value and how the TMLE-VIM(λ) p-values are returned. To choose λ data adaptively, we can repeat the whole procedure at different λ values, and select the λ associated with the lowest cross validated risk. p_{kj} is the TMLE p-value for j-th variable V_j and k-th correlation cut-off δ_k; W is the adjustment set from corresponding δ_k; Φ represents empty set.

Simulation II Data Generation

Each dataset in simulation II contains 1000 variables. What follows is a step-by-step explanation of how we generated our data. Each observation (Y_i, A_i, W_i) is indexed by i, $i = 1, \cdots, n$.

1. For W, jointly draw an $n \times 800$ matrix from a multivariate normal distribution with mean 0 (0.3 for polynomial model to create correlation between W and W^2) and covariance matrix S. The S was the correlation matrix computed from the top 800 ranked genes from **?**. The absolute median of S is around 0.26. For the rest 200 variables, each denoted by A_k, do the following:

2. For the k-th A, randomly sample 30 variables from W, and the k-th A is the sum of these 30 Ws added with a normal error e, $e \sim N(0, 5)$. This results in a median absolute correlation coefficient of 0.3 among As.

3. Standardize A_k (A_k^2 for the polynomial model) by its mean and standard error and so every A (A^2) has mean 0 and standard error 1.

4. Compute the outcome Y using 20 randomly sampled As. For the linear model:

$$Y = \sum_{k=1}^{20} \beta_k A_k + e,$$

   where β is $(2, -2, 2, -2, -2, -2, -2, -2, -2, 2, -2, -2, 2, -2, 2, -2, 2, 2, 2, 2)$ and $e \sim N(0, 5)$. For the polynomial model:

$$Y = 2 \sum_{k=1}^{20} \beta_k A_k^2 + e$$

5. Standardize all variables including all As and Ws by their means and standard errors.
