# Supplementary material for: Dimension reduction with gene expression data using targeted variable importance measurement
Source: BMC Bioinformatics. 2011 Jul 29;12:312. doi: 10.1186/1471-2105-12-312 (PMC3166941; doi:10.1186/1471-2105-12-312)
Supplement: Additional file 2 — The additional materials of the conducted simulations. [file 1471-2105-12-312-S2.PDF]

Additional materials for Simulation I

the mean prediction risks, the mean true positive and false positive counts and their standard errors

			n_w=250				n_w=500			
			UR-VIM		TMLE-VIM		UR-VIM		TMLE-VIM	
			mean	s.e	mean	s.e	mean	s.e	mean	s.e
		ρ								
MVR prediction risk	Testing set (a)	0.1	35.3375	6.3527	27.0652	0.6925	44.9756	6.2789	26.8271	0.548
		0.3	34.651	6.3336	27.0194	0.5462	35.4751	3.4696	27.1687	0.9484
		0.5	36.4017	3.9403	27.5728	1.1128	54.4314	8.1606	27.8516	1.029
		0.7	42.8094	3.7525	27.4038	1.4634	147.2843	40.5983	29.4385	2.557
		0.9	52.0526	5.3472	40.28	6.7052	1015.551	585.7472	61.9795	14.4908
	Testing set (b)	0.1	35.0634	6.1172	27.1723	0.7373	45.4216	6.1579	28.2331	0.5709
		0.3	35.5088	6.8729	27.351	0.5698	37.772	3.4897	29.0599	1.0016
		0.5	41.6521	5.3245	28.6782	1.5873	72.1494	13.3107	29.1528	1.2282
		0.7	75.534	10.8908	31.1804	3.421	370.7501	118.1705	33.615	4.9006
		0.9	229.9943	29.6305	63.3056	12.9242	8492.796	4996.916	97.6558	13.1937
D/S/A prediction risk	Testing set (a)	0.1	35.0751	6.424	26.5314	0.5118	44.827	6.1834	25.8633	0.2706
		0.3	33.7745	6.6059	26.2399	0.2707	32.4379	3.394	25.8619	0.3291
		0.5	30.5536	3.8114	26.9833	1.9531	31.4318	4.3194	27.4453	1.2974
		0.7	30.5141	2.9873	26.5251	1.8768	35.1508	4.9008	29.0355	1.9612
		0.9	35.5113	2.4107	40.3567	7.0233	39.8768	2.1859	61.8023	14.841
	Testing set (b)	0.1	34.8486	6.1899	26.6567	0.5136	45.0855	6.2011	27.3101	0.2899
		0.3	34.1768	6.9805	26.4972	0.2184	34.2449	3.6522	27.4807	0.375
		0.5	31.4305	3.8861	27.3918	1.7848	32.6179	4.2379	28.6486	2.3691
		0.7	38.6117	7.0031	28.785	4.4136	46.804	12.3414	32.8769	4.4442
		0.9	87.7662	11.4019	63.1434	13.8379	129.6179	15.102	95.1835	15.1649
	Number of identified As in the reduced candidate list	0.1	23	1.4142	25	0	20.6	1.5055	25	0
		0.3	23.2	1.6193	25	0	23.4	0.8433	25	0
		0.5	24.1	0.8756	25	0	24.2	0.9189	25	0
		0.7	24.8	0.4216	25	0	24.6	0.5164	24.6	0.6992
		0.9	24.6	0.5164	20.7	1.767	24.9	0.3162	13.7	3.199
	Number of identified Ws in the reduced candidate list	0.1	2.3	1.6364	1.5	1.8409	2.6	1.4298	2.3	0.9487
		0.3	14.4	6.0406	2.2	1.6865	40.7	18.4394	3.9	3.3813
		0.5	101.4	15.6361	3.6	3.2728	228.5	29.4816	3.4	2.1705
		0.7	207.1	15.1177	5.7	3.4335	400.8	22.5379	6.5	3.2059
		0.9	238.8	6.7791	8.7	3.335	486	8.9318	9.9	4.9989
	Number of identified As in the D/S/A prediction model	0.1	23	1.4142	25	0	20.6	1.5055	25	0
		0.3	23.2	1.6193	25	0	23.4	0.8433	25	0
		0.5	24	0.8165	24.9	0.3162	23.9	0.8756	24.8	0.4216
		0.7	23.1	1.1005	24.7	0.6749	21.8	2.044	24.1	0.7379
		0.9	15.9	1.8529	19.6	1.9551	9.9	2.2828	13.2	3.3267
	Number of identified Ws in the D/S/A predicition model	0.1	0.3	0.483	0	0	1.4	1.075	0	0
		0.3	0.8	0.6325	0	0	0.7	0.6749	0	0
		0.5	0.7	0.6749	0.1	0.3162	1	0.8165	0.2	0.4216
		0.7	1.9	1.1005	0.3	0.6749	3.2	2.044	0.9	0.7379
		0.9	9.1	1.8529	4.8	1.8135	15.1	2.2828	7.5	2.7988

Additional materials for Simulation II

the mean prediction risks, the mean true positive and false positive counts and their standard errors

		Simulation Type							
		Linear				Polynomial			
		p-value < 0.05		top 100		p-value < 0.05		top 100	
		mean	s.e.	mean	s.e.	mean	s.e.	mean	s.e.
Prediction risk	UR-VIM	64.4247	10.575	77.5007	11.067	241.322	54.413	270.6122	28.6659
	TMLE-VIM(Q_0=UR)	46.6574	14.9514	73.3044	30.658	200.053	28.7677	248.6219	35.6491
	TMLE-VIM(Q_0=LASSO)	33.6119	3.7104	35.7003	2.6512	171.6834	18.788	173.7704	14.6754
	TMLE-VIM(λ)	32.0081	3.0362	36.9127	3.5208	163.0085	18.775	181.6062	29.6803
Number of true positives (As) in the reduced candidate list	UR-VIM	13.8	1.99	9	2.21	13.4	2.27	8.2	2.78
	TMLE-VIM(Q_0=UR)	16.6	1.78	8.8	5.85	14.7	1.49	7.2	5.43
	TMLE-VIM(Q_0=LASSO)	19.7	0.48	19.9	0.32	17.9	1.52	19.2	1.23
	TMLE-VIM(λ)	20	0	20	0	19.2	1.32	17.7	4.03
Number of false positives (Ws) in the reduced candidate list	UR-VIM	605.3	212.43	na	na	555.9	261.7	na	na
	TMLE-VIM(Q_0=UR)	280.5	151.72	na	na	255.5	123.01	na	na
	TMLE-VIM(Q_0=LASSO)	29.1	12.45	na	na	24	9.5	na	na
	TMLE-VIM(λ)	41.6	31.52	na	na	105.9	88.26	na	na
