# Supplementary material for: Dimension reduction with gene expression data using targeted variable importance measurement
Source: BMC Bioinformatics. 2011 Jul 29;12:312. doi: 10.1186/1471-2105-12-312 (PMC3166941; doi:10.1186/1471-2105-12-312)
Supplement: Additional file 3 — The PCA results. [file 1471-2105-12-312-S3.PDF]

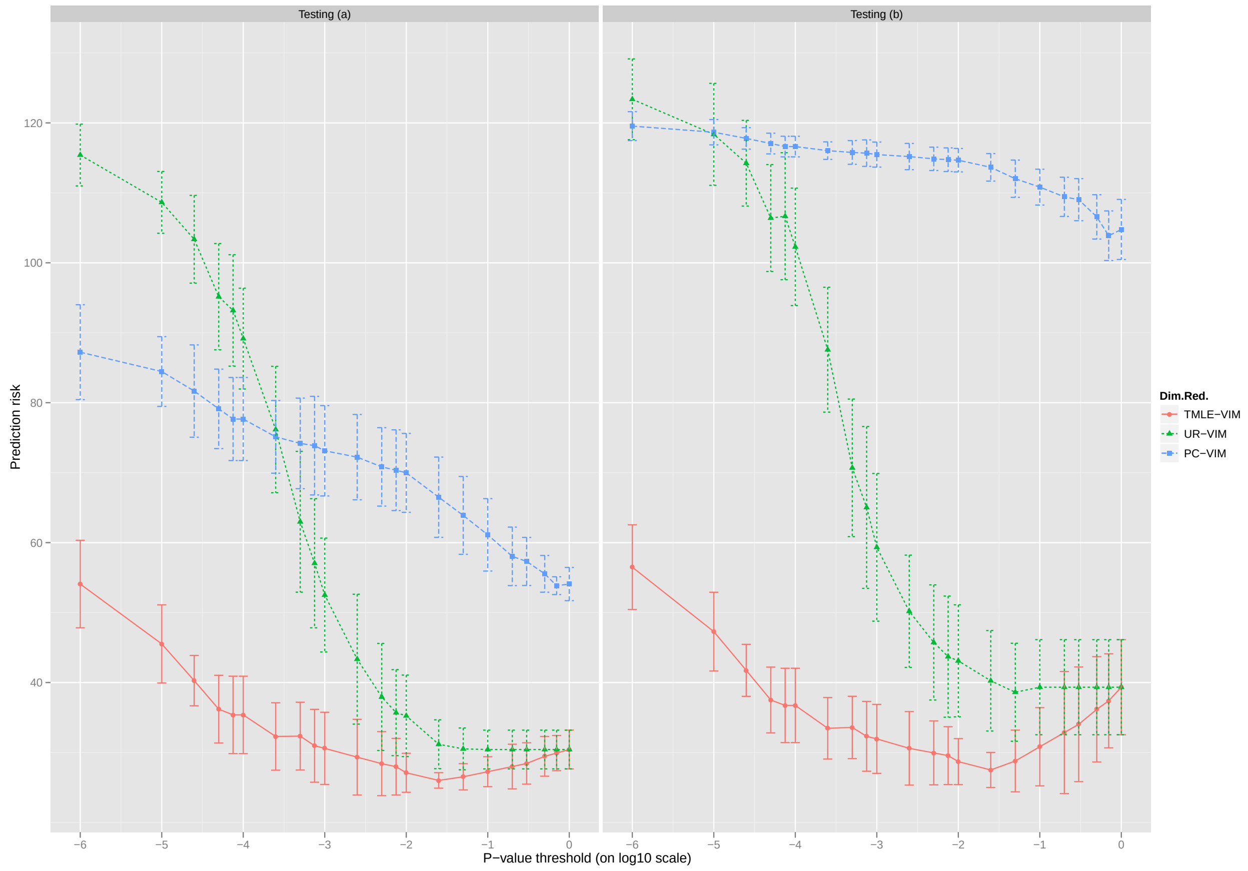

Figure 1: The prediction risk from the UR-VIM, the TMLE-VIM and the PCA for the simulation I data with setup $(m_w = 10, \rho = 0.7, \sigma_e = 5)$. D/S/A risks are plotted across a range of p-value thresholds that were used to truncate the variable (or component in the case of PCA) list in a dimension reduction procedure. The left panel plots prediction risks calculated on the testing set (a), and the right panel on the testing set (b). Red dots represent the TMLE-VIM, green triangles represent the UR-VIM, and blue squres represent the PCA. Standard error bars are superimposed on each point.
